# Fatal Disseminated *Cryptococcus gattii* Infection in New Mexico

**DOI:** 10.1371/journal.pone.0028625

**Published:** 2011-12-14

**Authors:** Carla J. Walraven, Wendy Gerstein, Sarah E. Hardison, Floyd Wormley, Shawn R. Lockhart, Julie R. Harris, Annette Fothergill, Brian Wickes, Julie Gober-Wilcox, Larry Massie, T. S. Neil Ku, Carolina Firacative, Wieland Meyer, Samuel A. Lee

**Affiliations:** 1 Section of Infectious Diseases, New Mexico Veterans Healthcare System, Albuquerque, New Mexico, United States of America; 2 Division of Infectious Diseases, University of New Mexico Health Science Center, Albuquerque, New Mexico, United States of America; 3 Department of Biology, South Texas Center for Emerging Infectious Diseases, The University of Texas at San Antonio, San Antonio, Texas, United States of America; 4 Mycotic Diseases Branch, Centers for Disease Control and Prevention, Atlanta, Georgia, United States of America; 5 Department of Microbiology and Immunology, University of Texas Health Science Center, San Antonio, Texas, United States of America; 6 Molecular Mycology Research Laboratory, Centre for Infectious Diseases and Microbiology, Westmead Millennium Institute, Sydney Medical School - Westmead Hospital, The University of Sydney, New South Wales, Australia; Louisiana State University, United States of America

## Abstract

We report a case of fatal disseminated infection with *Cryptococcus gattii* in a patient from New Mexico. The patient had no history of recent travel to known *C. gattii-*endemic areas. Multilocus sequence typing revealed that the isolate belonged to the major molecular type VGIII. Virulence studies in a mouse pulmonary model of infection demonstrated that the strain was less virulent than other *C. gattii* strains. This represents the first documented case of *C. gattii* likely acquired in New Mexico.

## Introduction

*Cryptococcus gattii* was first isolated from a pediatric patient in the Congo [1], and environmentally from various species of *Eucalyptus* trees in Australia, where rural aboriginals have frequently been reported with infection [2]. It has since been reported from eucalyptus and other trees in tropical and subtropical regions including southern California, South America, parts of Africa and Southeast Asia [3], [4]. Before 1999, *C. gattii* infections were only rarely reported from temperate climates of North America [5], [6]; however, since 1999, an outbreak of *C. gattii* infections has been reported from British Columbia (BC), and more recently from the U.S. Pacific Northwest (Washington and Oregon) [7]–[9]. Although *C. gattii* was previously believed to be a tropical fungus, these outbreaks demonstrate a larger ecological niche. Investigations into the source of the outbreak in the temperate climate of Vancouver Island and the Pacific Northwest (PNW) provide evidence that decaying wood from tree hollows, soil, fresh and saltwater may all be sources of *C. gattii* [8]–[10].

*C. neoformans* and *C. gattii* are the only two major species of the genus *Cryptococcus* considered to be pathogenic in humans. Prior to the AIDS epidemic, cryptococcal infections were rare. Since the molecular characterization of *C. neoformans* and *C. gattii* and classification into separate species, ecological and clinical differences have been reported. *C. gattii* tends to be seen clinically in immunocompetent patients, although the spectrum of infection can involve immunocompromised patients as well, whereas *C. neoformans* is primarily an opportunistic pathogen. Both *C. neoformans* and *C. gattii* have a propensity to cause pulmonary and central nervous system (CNS) disease. In patients from Papua New Guinea and Australia, *C. gattii* is more likely than *C. neoformans* to cause cryptococcomas in the lung and brain, which may be mistaken for malignancies (as in this case) or abscesses [11], [12]. Furthermore, whereas the outbreak strain identified in BC/PNW more commonly presents with a respiratory syndrome, non-outbreak strains appear to present more commonly with CNS infection [12], [13].

The onset of cryptococcal infection is often subacute and nonspecific, with headache being the most common presenting symptom [14]. Compared to infections with *C. neoformans*, patients with *C. gattii* cerebral involvement were found to have more severe neurological sequelae such as hydrocephalus and focal CNS findings, including ataxia, hearing loss, altered mentation, papilledema, and higher intracranial pressures often requiring surgical procedures for management. Infections due to *C. gattii* follow a prolonged clinical course and are slower to respond to antifungal therapy; however mortality does not appear to be higher than *C. neoformans* infections [15], [16].

We report the first documented case of *C. gattii* infection in New Mexico, from a patient without a recent history of travel to a known *C. gattii-*endemic area. Because the patient was immunocompetent and New Mexico is not currently known to be a *C. gattii*-endemic region, the infection went unrecognized until autopsy.

## Results

### Clinical Presentation

The patient was a 56-year-old Hispanic man who was brought to the emergency room (ER) in late April 2010 for evaluation of a 2.5-month history of intermittent, progressively worsening headache. The patient was noted to have mild confusion. He reported no fever, chills, dyspnea, nausea, vomiting or diarrhea. He had returned home in February 2010 after release from a two-month incarceration in a state prison in Santa Rosa, NM. Upon entering his home, he found that his dogs had expired, and his house was extensively contaminated with dog feces. Several days after cleaning his house, he reported experiencing a headache which worsened over the next 2.5 months. In the ER, the patient was afebrile and his physical and neurological exam was unremarkable. Initial serum laboratory tests indicated mild leukocytosis (WBC 11.6×10^9^/L, normal range 4–10.6), and hyponatremia (sodium 128 mmol/L, normal range 137–145). A chest radiograph showed a focal infiltrate or mass estimated to be 4×8 cm in the lingula as well as additional perihilar infiltrates, particularly in the right upper lobe. A computed tomography of the head without contrast revealed no evidence of focal lesions, and an opacified left maxillary sinus suggestive of sinusitis. The patient was begun on empiric ceftriaxone and azithromycin for possible community-acquired pneumonia, and admitted for further evaluation of headache and confusion.

The patient's prior medical history included hypertension, diabetes, and depression. His medication list included lisinopril, naproxen, hydrocodone/acetaminophen, and lorazepam. His family history was unremarkable. The patient had a one-year history of smoking but had quit in December 2009. He drank alcohol rarely and denied the use of recreational drugs. The patient was born and lived alone in the same house which was located in the South Valley area of Albuquerque, NM. In his home, he was reported to have burned firewood for heat. There was a chicken coop on his property where a relative raised chickens. He was employed as a sanitation truck driver. The patient rarely traveled, other than the recent incarceration in Santa Rosa, NM, and has never left the state of New Mexico. The patient did note a 30-pound weight loss during his two months of incarceration.

Further testing included HIV antibody ELISA, which was negative; CD_4_ T-cell count was 560 cells/mm^3^. Magnetic resonance imaging of the brain revealed a sub-centimeter focus of restricted diffusion in the left global pallidus that was consistent with acute or sub-acute ischemic infarct, and a focus of abnormal increased T2 signal intensity in the sub-cortical white matter. A chest CT revealed the previously noted lingular mass, measuring 5.5×4.5 cm. Bilateral patchy multifocal nodular densities were identified in bilateral upper and lower lobes, some of which had a ground glass appearance. No significant adenopathy was identified. A PET-CT scan revealed a metabolically active mass in the lingual; clinically suspected to be a malignancy. During the course of the patient's initial hospitalization of six days, the patient's mental status, hyponatremia, and overall clinical status improved. Further evaluation of the lung mass was planned as part of an outpatient management plan.

Ten days later, the patient was brought back to the emergency room for evaluation of altered mental status. Upon arrival to the ER, the patient was initially responsive only to noxious stimuli and was found to be severely hypertensive (BP 201/126) and tachycardic (P 129). He responded to naloxone and labetalol. On exam, pupils were noted to be initially unequal (right 2 mm, left 4 mm) but were reported equal after administration of naloxone. After becoming more alert, the patient stated that he had developed progressive, severe weakness over the past 3 weeks resulting in difficulty lifting his extremities. He was essentially bed-bound as a result. Furthermore, he reported intermittent, severe headache with associated neck pain. The patient also noted “ringing in his ears” for the preceding four days, with difficulty hearing anything else.

A portable chest radiograph revealed no change in the lingular mass and new scattered areas of patchy infiltrate in both lung fields. A head CT without contrast was unchanged from the prior CT. Empiric intravenous vancomycin and piperacillin-tazobactam were given after the collection of blood cultures for treatment of possible healthcare-associated pneumonia. A repeat brain MRI the next day was also unchanged. Empiric azithromycin was added on day two of the patient's hospitalization. The patient was found unresponsive on hospital day two and expired shortly thereafter.

### Microbiologic and Pathological Examination Identifies *C. gattii*

**Figure 1:**
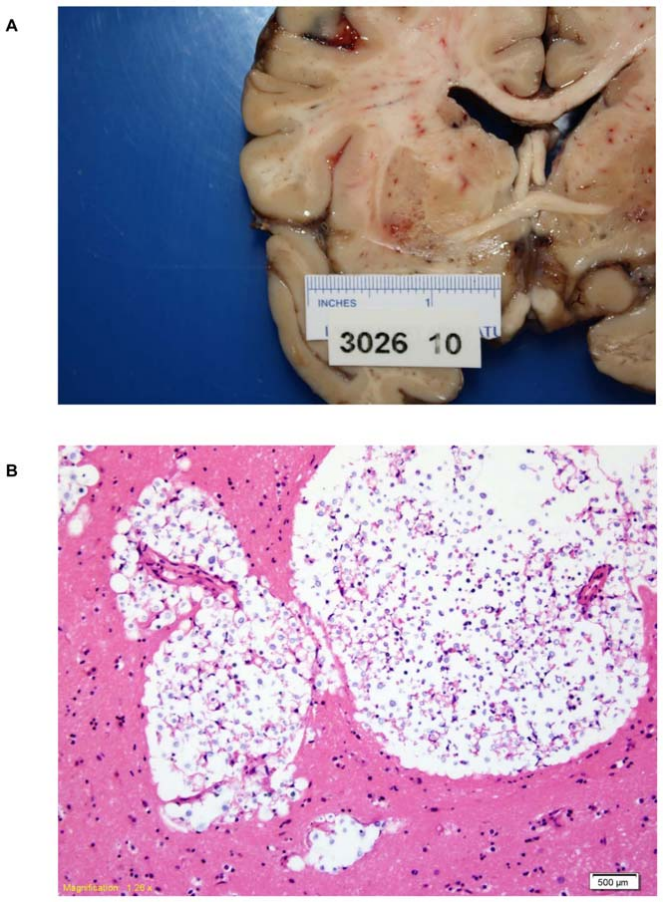
Postmortem examination of the brain demonstrates cryptococcal lesions. **a.** On gross examination, sections through the cerebral hemispheres demonstrated multiple, small vesicular lesions predominantly in the bilateral basal ganglia and additional lesions scattered throughout the bilateral white matter tracts. No lesions were present in the cerebellum or brainstem. **b.** On histopathologic exam, hemotoxylin/eosin stained sections of the vesicular lesions showed diffuse clusters of yeast cells.

A post-mortem examination was performed. Sections through the cerebral hemispheres demonstrated multiple, small vesicular lesions predominantly in the bilateral basal ganglia and also scattered throughout the bilateral white matter tracts (Figure 1A). No lesions were present in the cerebellum or brainstem. On histologic exam, sections from the bilateral basal ganglia showed multiple, cleared-out spaces containing numerous round yeast forms (Figfure 1B).

**Figure 2:**
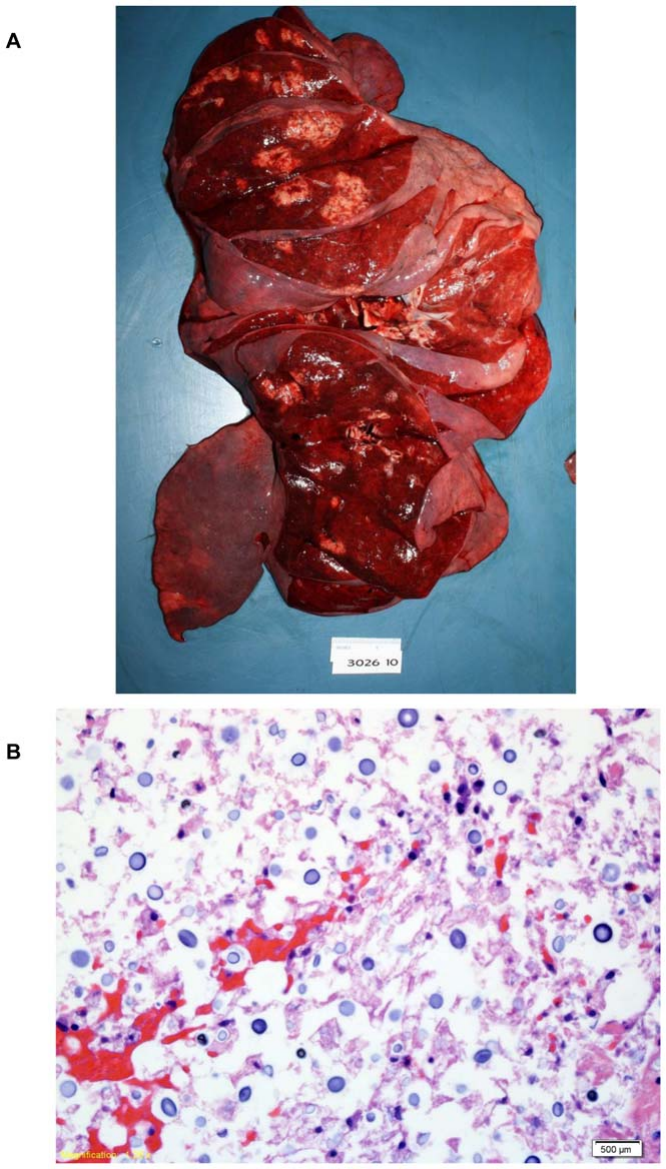
Postmortem examination of the lungs reveals multiple cryptococcal lesions. **a.** Postmortem examination of the lungs revealed multiple yellow-tan ill-defined masses throughout all lobes of the lungs. **b.** Histopathologic examination of the lungs demonstrated scattered interstitial and intravascular encapsulated yeasts morphologically consistent with *Cryptococcus* species.

In the lungs, a 7.5×5.0×4.0 cm, well-circumscribed yellow-tan mass was present in the lingula. Diffuse, bilateral, tan, ill-defined lesions were also present in all lobes of the lungs (Fig. 2A). Lung sections revealed occasional scattered, interstitial and intravascular clusters of encapsulated round yeasts morphologically consistent with *Cryptococcus* species (Figure 2B). Significant pulmonary edema with scattered acute inflammatory cells and macrophages were also present. Gomori methenamine silver (GMS) and mucicarmine stains of lung tissue revealed scattered yeasts.

Blood cultures collected on admission to the hospital as well as postmortem bacterial and fungal cultures of the lungs and heart blood grew *Cryptococcus* species, identified as *C. gattii* by the Fungus Testing Laboratory (University of Texas Health Science Center, San Antonio) on June 23, 2010.

### Multilocus Sequence Typing (MLST) Identifies the VGIII Molecular Genotype

**Figure 3:**
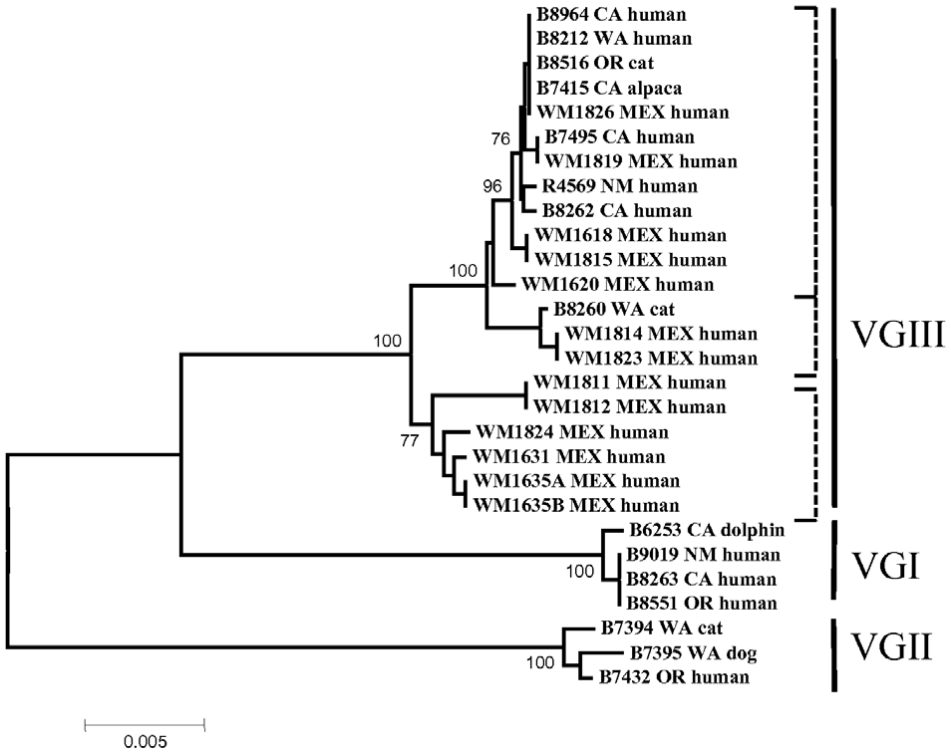
Neighbor joining dendrogram showing genotype VGIII isolates from the Pacific Nothwest of the US, California and Mexico. The isolate from New Mexico is more closely related to isolates from California, Washington and Oregon than to most of the isolates from Mexico, possibly indicating a US enriched clade of *C. gattii* VGIII. Significant bootstrap values are shown.

To delineate the relationship between the clinical isolate R4569 and isolates from the *C. gattii* emergence in the Pacific Northwest U.S. and British Columbia (PNW), MLST analysis, using the seven loci of the consensus MLST typing scheme was performed [17]. The isolate R4569 was genotyped as belonging to the major molecular type VGIII, which is different from the VGII major molecular type of the majority of the emerging isolates from the Pacific Northwest. To more accurately pinpoint the origin of isolate R4569, the phylogenetic relationships between a number of reference *C. gattii* isolates from the U.S. and neighboring Mexico were analyzed and a neighbor-joining dendrogram was constructed based on the seven concatenated MLST sequences (Figure 3, Table 1). The VGIII isolates investigated fell into three distinct clades, one containing isolates exclusively from Mexico, and two containing a mixture of Mexican and U.S. isolates. The New Mexico isolate (R4569) was more closely related to U.S. isolates and this grouping had a high bootstrap value.

**Table 1:** Isolates used for comparison in this study.

		MLST allele									
Isolate	State	CAP59	GPD1	LAC1	PLB1	SOD1	URA5	IGS1	Source	Subtype	Reference
B7415	CA	18	3	3	6	40	19	1	alpaca	VGIII	29
B8964	CA	18	3	3	6	40	19	1	human	VGIII	This study
B8212	WA	18	3	3	6	40	19	1	human	VGIII	29
B8516	OR	18	3	3	6	40	19	1	cat	VGIII	This study
R4569	NM	18	3	3	20	28	25	1	human	VGIII	This study
B7495	CA	18	3	32	20	40	23	1	human	VGIII	29
B8260	WA	29	9	2	4	28	19	18	cat	VGIII	29
WM 1826	Mexico	18	3	3	6	40	19	1	human	VGIII	43
B8262	CA	18	3	3	34	38	19	1	human	VGIII	29
WM 1815	Mexico	18	3	22	6	28	19	1	human	VGIII	43
WM 1819	Mexico	18	3	32	20	40	23	1	human	VGIII	43
WM 1814	Mexico	29	7	2	4	28	21	5	human	VGIII	43
WM 1823	Mexico	29	7	2	4	28	21	5	human	VGIII	43
WM 1618	Mexico	18	3	22	6	28	19	1	human	VGIII	43
WM 1635	Mexico	18	3	22	21	32	19	1	human	VGIII	43
WM 1620	Mexico	18	3	20	17	28	29	23	human	VGIII	43
WM 1824	Mexico	18	3	20	17	32	29	1	human	VGIII	43
WM 1631	Mexico	18	3	22	17	32	19	23	human	VGIII	43
WM 1811	Mexico	35	3	22	20	41	28	5	human	VGIII	43
WM 1812	Mexico	35	3	22	20	41	28	5	human	VGIII	43
B6253	CA	16	15	5	5	45	12	3	dolphin	VGI	44
B8263	CA	16	5	5	5	32	12	3	human	VGI	29
B8551	OR	16	14	5	5	32	12	3	human	VGI	This study
B7394	WA	2	6	4	2	15	2	10	cat	VGIIb	29
B7395	WA	1	1	4	1	14	7	4	dog	VGIIa	29
B7432	OR	6	6	4	1	15	2	15	human	VGIIc	29

### R4569 is Less Virulent in Mice than Other Known *C. gattii* Strains

**Figure 4:**
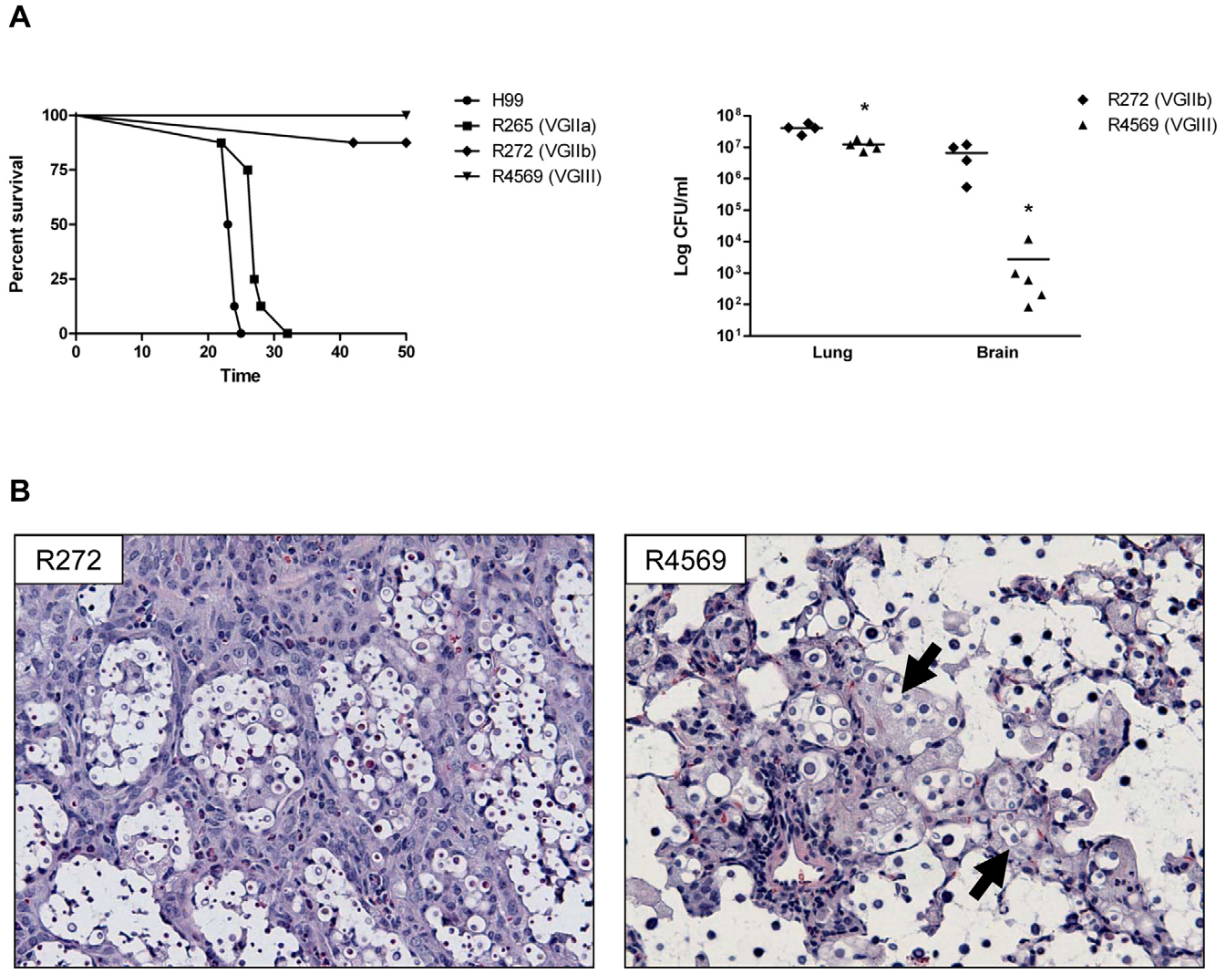
Strain R4569 is hypovirulent compared to VGII strains R265 and R272. **a.** Eight mice per group were intranasally inoculated with 1×10^5^ CFU of each strain and monitored for disease. Mice infected with *C. neoformans* strain H99 or *C. gattii* strain R265 had mean survival times of 23.5 and 27 days, respectively. 87.5% of mice survived infection with R272, and all mice infected with R4569 survived. Survival curves between R272 and R4569 were not significantly different, but fungal burden determination at day 50 post-infection revealed significantly less yeast in the lungs and brains of mice infected with R4569 (*p*<0.05, Student's *t*-test). **b.** Sections of lung from mice infected with R272 show greater numbers of organisms as compared to infections from R4569. There are also increased numbers of lymphocytes and macrophages around aggregates of yeast with R272 than observed with R4569. Additionally, yeasts are found within “foamy” macrophages with R4569 (arrows) suggesting greater control of the infection.

To assess the virulence potential of the R4569 isolate in an animal model, BALB/c mice were intranasally infected with 1×10^5^ CFU of R4569. Separate cohorts of mice were also infected with *C. gattii* strains R265 and R272, reference strains for VGIIa and VGIIb molecular subtypes respectively, associated with the Vancouver Island outbreak [18], as well as the *C. neoformans* reference strain H99 [19]. All mice infected with *C. gattii* strain R265 and *C. neoformans* strain H99 succumbed to infection with median survival times of 27 days and 23.5 days, respectively (Figure 4a) similar to previous studies [20], [21]. Survival rates for mice infected with *C. gattii* strains R272 and R4569 were significantly greater (87.5% and 100% survival, *p*<0.05) than mice infected with R265 or H99 (Figure 4a). The survival rate of mice challenged with *C. gattii* R272 strain was similar to earlier survival analysis using this strain [21] (Wormley lab, unpublished observations). Nonetheless, no mice infected with strain R4569 appeared ill by day 50 post-infection; however, fungal burden determination from the lungs and brain of mice at the termination of the experiment revealed the presence of numerous viable yeasts in mice infected with strain R272 or R4569 (Figure 4a). Significantly fewer CFUs were cultured from the lungs and brains of mice infected with strain R4569 than those infected with strain R272. Furthermore, histological examination revealed greater inflammation in the lungs of mice infected with strain R272 compared to strain R4569 (Figure 4b). Many yeast cells of strain R4569 were confined within activated “foamy” macrophages, that are indicative of increased intracellular killing of cryptococci by macrophages and thus control of fungal replication (Figure 4b, arrows) [22], [23]. Altogether these results suggest that strain R4569 is less virulent than the VGII reference isolates in a mouse model of pulmonary infection or, alternatively, an enhanced capacity of mice to limit the growth of R4569 yeast.

## Discussion

Our findings represent the first documented case of *C. gattii* infection believed to have been acquired in New Mexico. The source of *C. gattii* infection in this patient remains unknown. The patient had no travel history to a known endemic region, and while *C. gattii* infection in domestic animals has been documented, these have been reported in animals residing in areas endemic to *C. gattii* [8], [24]. New Mexico is an arid region covered mostly by mountains, plains, and desert, with little annual rainfall and relatively low humidity. Since 2002, there has been an increase in precipitation around the Albuquerque area, which was highest between 2004 and 2008 compared to the 30-year normal averages. In addition, the annual temperature has also been above average, with a median temperature of 58 degrees Fahrenheit [25]. New Mexico has regions similar to those in Mexico known to harbor *C. gattii* [26]–[29], whereas the foothills of the Rocky Mountains, which run through Albuquerque, may represent a more temperate climate niche. Environmental sampling in the Pacific Northwest demonstrated that *C. gattii* has established ecological niches in trees other than *Eucalyptus*, including species of fir, maple, alder, cedar, spruce, pine and oak trees [10], [24]. In another environmental surveillance study, certain species of cacti in Puerto Rico were also found to harbor *C. gattii* [4], [30]. Both of these sources could potentially serve as an ecological niche for *C. gattii* in New Mexico. Further environmental surveillance of the patient's residential area was not conducted (as resources to pursue this are not available).

In general, cryptococcal disease appears to be rare in New Mexico. Between 2003 and 2007, we estimate there were at least one to two confirmed cases of cryptococcal disease in the Albuquerque area, which is home to the four largest hospitals in New Mexico. Starting in 2008, the number of confirmed cases increased with three cases in 2008, eight cases in 2009 and at least five cases to date in 2010. The autopsy report of this patient led to the diagnosis of disseminated *C. gattii* infection. However, due to the initial clinical impression of lung malignancy, the patient's immunocompetent status and lack of a travel history did not heighten clinical awareness of a cryptococcal infection in time to initiate antifungal therapy. Because the clinical presentation was most suggestive of a lung mass or malignancy, and the actual diagnosis is infrequently seen in this area, further testing for invasive fungal infection was not performed. The patient had widely disseminated infection with extensive inflammation, and likely succumbed to overwhelming systemic infection and sepsis, rather than elevated intracranial pressure with brainstem herniation, based on post-mortem findings. To date, *C. gattii* infections in the United States have been reported in Washington, Oregon, California, and North Carolina, in which all patients had some geographical exposure to endemic regions. [7], [8], [31], [32] The case reported here is similar to another case report of a 44-year-old immunocompetent man in Japan with *C. gattii* CNS disease who had no history of recent overseas travel [33]. Although this strain was VGIIa, similarities to this case include the endemic nature of the infection, and clinically, the mass lesion was also initially suspected to be a tumor. Cases such as these, in which immunocompetent patients who lack geographical risk factors living in non-endemic areas are acquiring *C. gattii* infections, suggest that there might be a broader distribution of the pathogen than is currently recognized.

The non-VGII genotype of this *C. gattii* isolate indicates it does not represent the migration of the PNW outbreak strains southward to New Mexico. Because of the lack of a clonal relationship to isolates from Mexico, it also does not likely represent the recent migration of *C. gattii* isolates northward from Mexico. Rather, this isolate likely represents the emergence of a clonal genotype that, based on geography of the common isolates has probably persisted in the U.S. for quite some time. A recent literature review [24] reported that VGIII isolates of *C. gattii* are most common in South America but they are also found in North America, Central America, Australasia and Southeast Asia. It was also recently reported that the majority of *C. gattii* isolates from HIV+ patients in California are VGIII [31]. Isolates of the genotype VGIII have not been found so far in environmental samples from British Columbia [10] nor from the U.S. PNW (S. Lockhart, unpublished results) but VGIII environmental isolates have been infrequently found in the U.S. in association with eucalyptus trees [34]. Subtype VGIII isolates had not been reported from the PNW emergence until a single isolate was reported from Washington in 2009 [18], [32]. Subsequently, human and veterinary isolates were reported from Washington, Oregon, and California, dating back to as early as 1992 [35]. It will be important to monitor further for the emergence of this genotype in other parts of the southwestern U.S.

*C. gattii* causes granulomatous pulmonary and disseminated disease in mouse inhalation models of infection, irrespective of the mouse strain used. In terms of genotypic variation in virulence, VGIIa isolates from the U.S. and from the Vancouver outbreak are more virulent in mice than VGIIb isolates from either region [36]–[39]. Our results suggest that the VGIII isolate R4569 is less virulent than either of the VGII strains tested. The morbidity observed in mice given an experimental infection with the VGIII isolate R4569 is similar to the morbidity observed in mice experimentally infected with other VGIII clinical isolates. Of the *C. gattii* cases reported to the CDC between 2004 and 2010, 50% were molecular type VGIIa, 10% were VGIIb, and only 3% were VGIII [40]. While the relationship of mouse virulence to human disease is unclear, it has been demonstrated that *C. gattii* infections are immunologically similar in mice and infected humans with respect to cytokine profiles [37], [41]. *C. gattii* replicates intracellularly within macrophages. It has been demonstrated that intracellular replication rate within murine macrophages *in vitro* is correlated with *in vivo* virulence in mice [38], [42], [43]. VGIIa isolates replicate more quickly in macrophages *in vitro* and are more virulent *in vivo* than VGIIb isolates. Furthermore, intracellular replication rate in murine macrophages correlates very highly with the intracellular replication rate in human macrophages, suggesting there might be a correlation between genotype and human virulence and disease [43]. Additional studies comparing the intracellular growth rate of *C. gattii* R4569 and other isolates within murine and human macrophages will be needed to support this hypothesis. The major clinical point from these in vivo studies is that despite strain R4569 being less virulent in this mouse infection model is that it is still highly capable of causing severe morbidity and mortality, particularly in the absence of timely diagnosis and therapy.

Based on the few previous reports [18], [32], [35], [38] there may be a low level of *C. gattii* infection in the U.S. due to VGIII isolates that may be unrecognized outside of HIV+ patients. However, the underlying level of infection due to *C. gattii* is currently unknown because many of the cases of cryptococcosis in the U.S. are diagnosed using an antigen test and most cases outside of the Pacific Northwest are assumed to be caused by *C. neoformans.* The heterogeneity of the VGIII isolates in this study, as compared to the almost exclusively clonal nature of the current VGII PNW emergence isolates, would be consistent with a long-term presence in the U.S. and a possibly unrecognized, long term, low level, endemicity. It is also interesting to note that among HIV+ patients in California in the 1990's almost all of the infections due to *C. gattii* were caused by isolates of the VGIII genotype [31], [44], where globally VGIII isolates were more often found in the non- immunocompromised patient population [45], [46]. We therefore support speciation of *Cryptococcus* clinical isolates due to the implications for public health and for understanding the epidemiological distribution of this fungal pathogen. In addition, there is epidemiological evidence that there are substantive differences in the clinical presentation of *C. gattii* and the course of disease compared to *C. neoformans* [13].

As infections due to *C. gattii* increase both in the United States and worldwide, epidemiologic surveillance will play an important role in identifying new ecological niches or reservoirs for this emerging fungal pathogen. Since not all clinical and reference laboratories are equipped to differentiate between *C. gattii* and *C. neoformans*, a heightened clinical awareness of the disease will be required to prompt further identification and genotyping to track the distribution of *C. gattii*.

## Materials and Methods

### Approvals and Ethics Statement

The authorized representative of the patient in this manuscript has given written informed consent (as outlined in the PLoS consent form) to publication of their case details. These animal experiments were approved by The University of Texas at San Antonio Institutional Animal Care and Use Committee (IACUC), approved protocol number MU021-11/1A2, and mice were handled according to IACUC guidelines. An IRB waiver was provided by the New Mexico Veterans Healthcare System Office of Research.

### Microbiologic Identification

Fungal identification was performed at the Fungus Testing Laboratory (University of Texas Health Science Center, San Antonio) using canavanine-glycine-bromthymol blue (CGB) agar and *URA5* sequencing as previously described [5].

### Multilocus Sequence Typing

The isolate was subtyped using The ISHAM consensus multilocus sequence typing (MLST) scheme [17]. The *URA5, IGS, PLB1, SOD1, GPD1, LAC1,* and *CAP59* gene fragments were amplified as described by Meyer and colleagues [17]. Briefly, isolates were grown on YPD plus 0.5% NaCl and DNA was isolated using the UltraClean DNA Isolation Kit as described by the manufacturer (MO BIO Laboratories, Carlsbad, CA). Gene fragments were amplified and sequenced in both directions using the primers of Meyer *et al* [17]. Allele and sequence types were assigned according to the *C. gattii* MLST database at *mlst.mycologylab.org*. The compiled sequences were compared to *C. gattii* sequences collected during the U.S. PNW surveillance (SRL) or during a previous surveillance in Mexico [W. Meyer, unpublished data]. Phylogenetic relationships, the neighbor-joining tree and bootstrap values were calculated using the Mega 4.1 software package [47]. All generated allele and sequence types of the seven genetic loci studied herein are accessible at the *C. gattii* MLST database at *mlst.mycologylab.org*.

### Mouse Pulmonary Model of Infection

Female BALB/c (H-2^d^) mice, four to six weeks of age (National Cancer Institute/Charles River Laboratories), were used throughout these studies. Mice were housed at The University of Texas at San Antonio Small Animal Laboratory vivarium and handled according to guidelines approved by the Institutional Animal Care and Use Committee.

Pulmonary cryptococcal infections were initiated by nasal inhalation as previously described [48]. Briefly, BALB/c mice (eight per group) were anesthetized with 2% isoflurane using a rodent anesthesia device (Eagle Eye Anesthesia, Jacksonville, FL) and then given an inoculum of 1×10^5^ colony forming units (CFU) of *C. neoformans* strain H99 or *C. gatti* strains R265 (VGIIa), R272 (VGIIb), or R4569 (isolate from this case) in 50 µl of sterile PBS introduced directly into the nares. The strains R265 and R272 were provided by Joseph Heitman (Duke University Medical Center, Durham, NC). The inocula used for nasal inhalation were verified by quantitative culture on yeast peptone dextrose (YPD) agar. The mice were fed *ad libitum* and monitored by inspection twice daily. Mice were either euthanized when moribund or at day 50 post-inoculation. For histopathology (three mice per group), lungs were transcardially perfused with PBS then inflated with 10% formalin via an intratracheal catheter. Lungs were tied off, dissected, and stored in formalin until paraffin embedding, sectioned, and stained by hematoxylin and eosin (Histology Laboratory at The University of Texas Health Science Center at San Antonio). For colony-forming unit (CFU) determination in surviving mice (four to five mice per group), lung tissues were excised using aseptic technique, homogenized in 1 ml of sterile PBS, and cultured by 1∶10 dilutions on YPD agar supplemented with chloramphenicol (Mediatech, Inc., Herndon, VA). CFUs were enumerated following incubation at 30°C for 48 h.
